# Impact of hereditary periodic fever syndromes and its change over time

**DOI:** 10.1186/1546-0096-13-S1-P46

**Published:** 2015-09-28

**Authors:** M Niewerth, T Kallinich, A Hospach, R Behrendes, A Thon, K Minden

**Affiliations:** 1 Deut. Rheuma-Forschungszentrum Berlin, Programmbereich Epidemiologie, Berlin, Germany; 2 Universitätsmedizin Berlin - Charité, Kinderklinik, Berlin, Germany; 3 Klinikum Stuttgart, Olgahospital, Stuttgart, Germany; 4 Kinderkrankenhaus St. Marien, Landshut, Germany; 5 Medizinische Hochschule Hannover, Kinderklinik, Hannover, Germany

## Introduction

Hereditary periodic fever syndromes (HPF) with their clinical inflammation and associated symptoms impair many aspects of affected children's and adolescents’ lives. Patients experience fever, pain and fatigue; they are restricted in their overall-wellbeing, functioning and participation. Little is known about the extent of the perceived burden of illness and its change over time.

## Objectives

To study the perceived burden and treatment of the HPF FMF, TRAPS and CAPS over 10 years.

## Patients and methods

Data from patients with genetically proven FMF, TRAPS and CAPS, who were recorded in the National Paediatric Rheumatologic Database (NPRD) in the years from 2004 to 2013 were used for this analysis. Temporal changes in patient-reported outcomes (e.g., overall wellbeing [NRS 0-10], pain [NRS 0-10], functional capacity [CHAQ], school attendance) and anti-inflammatory medication were evaluated.

## Results

Altogether, 819 cases with HPF were recorded between 2004 and 2013: 703 with FMF, 47 with TRAPS and 69 with CAPS. The number of HPF patients recorded per year increased from 98 in 2004 to 346 in 2013. Treatment of HPF patients changed over time. While in 2004 5% of HPF patients were on biologics, this applied to 13% 10 years later. In 2013, 2% of FMF, 22% of TRAPS and 72% of CAPS patients were treated with IL-1 blockers and 3% of FMF, 56% of TRAPS, and 6% of CAPS patients with TNF-blockers. On these drugs, 51% of all patients had an active disease (physician global >0) at documentation in 2013 in comparison to 48% in 2004.

Considering the whole HPF group, patients perceived health did not change over time. In 2013, 42% of patients had restrictions in overall wellbeing (NRS>0) in comparison to 38% in 2004. However, CAPS patients reported better health in 2013, with lower mean values for overall wellbeing (1.35), pain (1.6) and functional limitations (CHAQ 0.32) than in 2004 (mean values 3.25, 1.9 and 0.47, respectively). CAPS and TRAPS patients as well reported a higher burden of illness in comparison to FMF patients still in 2013. One in four patients of the whole HPF group had missed school or kindergarten within the 4 weeks prior to documentation, without change over time. However, the average number of days missed at school decreased from 10.2 in 2004 to 5.1 in 2013.

## Conclusion

HPF place a significant burden on the affected individuals. Measures beyond drug treatment seem necessary to ensure that patients can live a full life.

## Acknowledgements

Supported by a grant from the German Child Arthritis Foundation (Deutsche Kinderrheuma Stiftung)

